# P-curve won’t do your laundry, but it will distinguish replicable from non-replicable findings in observational research: Comment on Bruns & Ioannidis (2016)

**DOI:** 10.1371/journal.pone.0213454

**Published:** 2019-03-11

**Authors:** Uri Simonsohn, Leif D. Nelson, Joseph P. Simmons

**Affiliations:** 1 Behavioral Science, ESADE Business School, Ramon Llull University, Barcelona, Spain; 2 Marketing Department, Haas School of Business, University of California-Berkeley, Berkeley, California, United States of America; 3 Operations, Information and Decisions department, The Wharton School, University of Pennsylvania, Pennsylvania, United States of America; Public Library of Science, UNITED KINGDOM

## Abstract

*p*-curve, the distribution of significant *p*-values, can be analyzed to assess if the findings have evidential value, whether *p*-hacking and file-drawering can be ruled out as the sole explanations for them. Bruns and Ioannidis (2016) have proposed *p*-curve cannot examine evidential value with observational data. Their discussion confuses false-positive findings with confounded ones, failing to distinguish correlation from causation. We demonstrate this important distinction by showing that a confounded but real, hence replicable association, gun ownership and number of sexual partners, leads to a right-skewed *p*-curve, while a false-positive one, respondent ID number and trust in the supreme court, leads to a flat *p*-curve. *P*-curve can distinguish between replicable and non-replicable findings. The observational nature of the data is not consequential.

## Introduction

*P*-curve is the observed distribution of statistically significant *p*-values (*p* ≤ .05) testing the hypotheses of interest from a set of studies. The shape of that distribution diagnoses if the findings contain evidential value, telling us whether we can statistically rule out selective reporting of studies (file-drawering) and/or analyses (*p*-hacking) as the *sole* cause of those statistically significant findings [1]. In follow-up work we have extended *p*-curve uses to estimate underlying statistical power in a way that corrects for selective reporting [2], made *p*-curve more robust to errors and fraud [3], and applied it to the popular and controversial power-posing literature [4]. An online app makes it easy to use *p*-curve by copy-pasting test results into a simple form (http://www.p-curve.com/app).

In a nutshell, true findings produce right-skewed *p*-curves, ones containing more low (e.g., .01s) than high (e.g., .04s) statistically significant *p*-values, whereas null findings produce flat or left-skewed *p*-curves, with at least as many as many high (.04s) as low (.01s) significant *p*-values. Studies generating a statistically significantly right-skewed *p*-curve contain evidential value.

Bruns and Ioannidis [5] examined the performance of *p*-curve analysis when applied to observational data, concluding that “*p*-curves based on true effects and *p*-curves based on null-effects with *p*-hacking cannot be reliably distinguished” (abstract). This conclusion is incorrect. As demonstrated below, *p*-curve can, and does, reliably distinguish between null effects and non-null effects. The observational nature of the data does not affect *p*-curve’s performance.

Bruns & Ioannidis’s conclusion seems to arise from their imprecise use of terminology. Specifically, they treat a false-positive finding and a confounded finding as the same thing. But they are different things. The distinction is as straightforward as it is important.

### Confounded effects

A confounded effect of X on Y is real and replicable, but it arises because another (omitted) variable causes both X and Y. Therefore, with a confounded effect, X does not *cause* Y, but the relationship between X and Y is still real and replicable.

### False-positive effects

In contrast, a false-positive effect of X on Y is neither real nor replicable. The apparent association between X and Y is instead entirely the result of sampling error.

Confounded effects are real and replicable, while false-positive effects are neither. Those are big differences, but Bruns & Ioannidis conflate them. For example, they write “the estimated effect size may be different from zero due to an omitted-variable bias rather than due to a true effect.” (p. 3; emphasis added). But omitted-variable bias does not make a relationship untrue; it makes it un-*causal*.

Once we distinguish between confounded and false-positive effects we see that *p*-curve performs as it should: It separates replicable from non-replicable results. Replicable results, whether causal or not, lead to right-skewed *p*-curves. False-positive, non-replicable effects lead to flat or left-skewed *p*-curves. And, again, *p*-curve is indifferent as to whether those data come from observational or experimental investigations.

*P*-curve’s inability to identify causality—to distinguish causal vs. confounded relationships—is not a shortcoming of *p*-curve analysis. Or at least it is no more of a shortcoming than its inability to fold laundry or file income tax returns. Identifying causal relationships is not something we can reasonably expect *any* statistical test to do. Every single statistical tool available, parametric and non-parametric, frequentist and Bayesian, merely establishes relationships between variables. No statistical tool could possibly differentiate correlational from causal relationships. Criticizing *p*-curve for failing to differentiate causation from correlation is like criticizing a professor for being mortal.

When researchers try to assess causality through techniques such as instrumental variables, regression discontinuity, or randomized field experiments, they do so via superior designs, not via superior statistical tests. The Wald, t and F tests that are reported in papers that credibly establish causality are the same Wald, t and F tests reported in papers that do not credibly establish causality. Correlation is not causation. Confusing the two is human error, not tool error.

## Demonstrations

To demonstrate *p*-curve’s ability to distinguish between replicable and non-replicable findings in observational data we provide two examples that use data from the General Social Survey [6]. In the first example we examine a confounded association: shotgun owners have had more female sexual partners. The omitted variable is gender.

Male respondents, relative to female respondents, are more likely to report owning a shotgun. Male respondents, relative to female respondents, also report having had a greater number of sexual encounters with females. This fact produces the relationship between shotgun ownership and number of female sexual partners, as controlling for gender makes the relationship go away. Using the entire GSS, a regression of number of female partners on shotgun ownership leads to a t = 9.29 and it drops to t = .88 when controlling for gender. We are not claiming, of course, that the residual effect is exactly zero. To showcase how *p*-curve performs in the presence of a real but non-causal effect, we analyze the data without controlling for gender.

**Fig 1:**
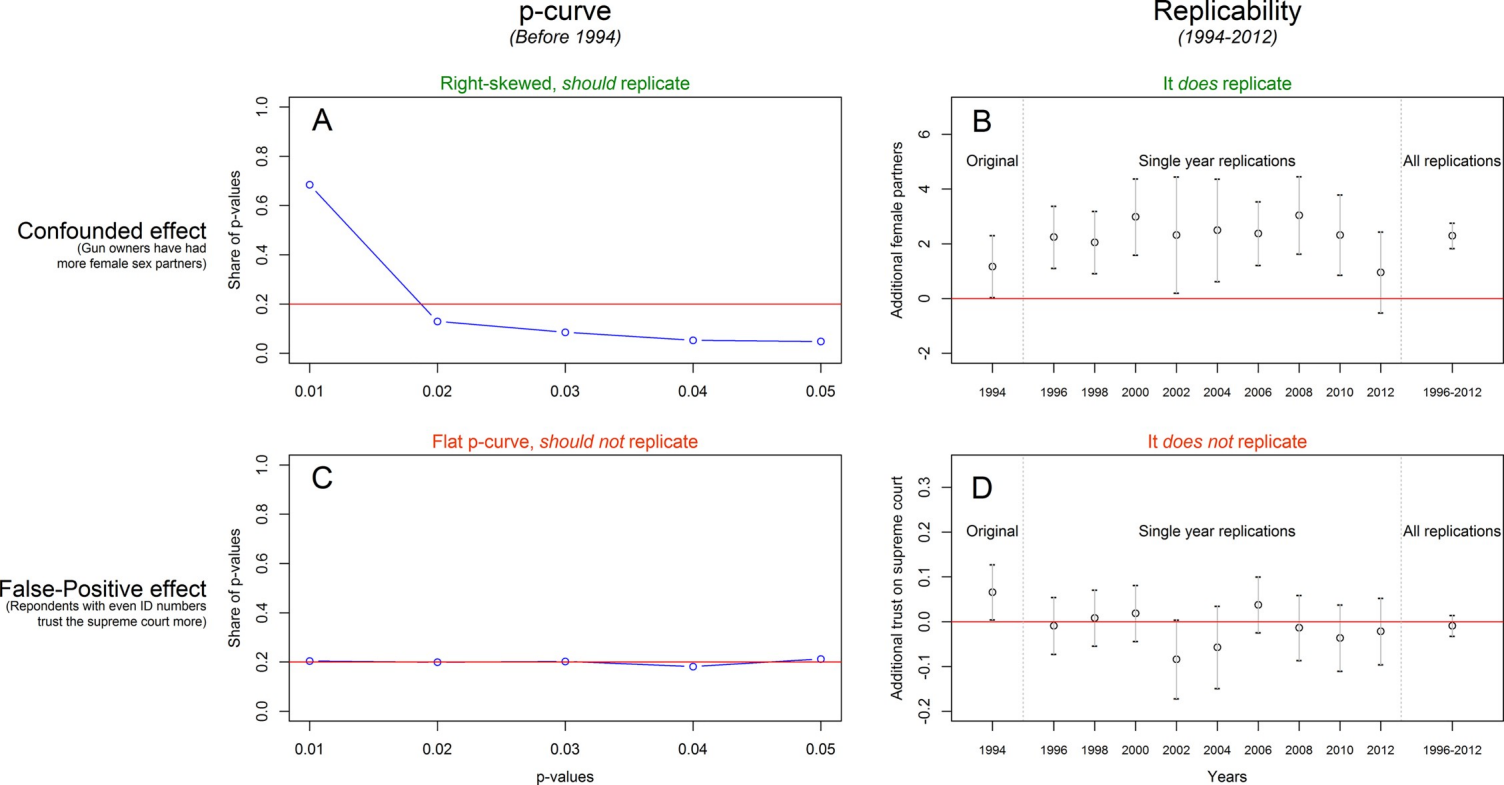
P-curve distinguishes between replicable and non-replicable findings. Notes: *P*-curves are obtained from bootstrapped samples with observations from years 1989–1993, using the sample size from the 1994 dataset. Reproduce figure: https://osf.io/qc43t/.

We created the original finding by analyzing the 1994 wave of the GSS, obtaining a significant relationship between shotgun ownership and number of female sexual partners. To construct a *p*-curve with multiple *p*-values we used data from previous years (1989–1993), following a procedure similar to Bruns and Ioannidis [5]. In particular, we generated random subsamples (of the size of the 1994 sample), re-ran the regression predicting number of female sexual partners with the shotgun ownership dummy, and constructed a *p*-curve for the subset of statistically significant results that were obtained. Simulations are not needed here as one can instead rely on the noncentral t-distribution (see e.g., Supplement 1 in Simonsohn et al., 2014b), but we did our best to follow the procedures by Bruns and Ioannidis (2016). Panel A in Fig 1 shows that this led to a right-skewed *p*-curve, which suggests that the finding should replicate in subsequent years. Panel B shows that it does.

Our second example involves a false-positive effect. With observational data it is difficult to identify effects that are truly zero because there is always the risk of omitted variables, selection bias, long and difficult-to-understand causal chains, etc.

To create a definitely false-positive finding in the GSS we started with a predictor variable that could not possibly be expected to truly correlate with any variable: whether the respondent was randomly assigned an odd vs. even identification number. We then *p*-hacked an effect by running t-tests on every other variable in the 1994 GSS dataset for odd vs. even participants, which produced 36 false-positive *p* < .05 results. For its amusement value, we focused on the question asking participants how much confidence they have on the U.S. Supreme Court (1: a great deal, 2: only some, 3: hardly any).

Panel C in Fig 1 shows that, following the same procedure as for the previous example, the *p*-curve for this finding is flat, suggesting that the finding would not replicate in subsequent years. Panel D shows that it does not. Fig 1 demonstrates how *p*-curve successfully distinguishes between statistically significant studies that are vs. are not expected to replicate. The observational nature of the data is not relevant.

P-curve works as it should.

## Conclusions

It is as important to distinguish causation form correlation when interpreting results from single studies, as it is when evaluating the performance of statistical procedures on sets of studies.

## References

[1] Simonsohn U, Nelson LD, Simmons JP. *p*-curve: A Key to the File Drawer. Journal of Experimental Psychology: General. 2014;143(2):534–47. 23855496 10.1037/a0033242

[2] Simonsohn U, Nelson LD, Simmons JP. P-Curve And Effect Size: Correcting or Publication Bias Using Only Significant Results. Perspectives on Psychological Science. 2014;9(6):666–81. 10.1177/1745691614553988 26186117

[3] Simonsohn U, Simmons JP, Nelson LD. Better P-Curves: Making P-Curve Analysis More Robust to Errors, Fraud, and Ambitious P-Hacking. Journal of Experimental Psychology: General. 2015;144(6):1146–52. 26595842 10.1037/xge0000104

[4] Simmons JP, Simonsohn U. Power Posing: P-Curving the Evidence. Unpublished manuscript2016. 10.1177/0956797616658563 28485698

[5] Bruns SB, Ioannidis JP. p-Curve and p-Hacking in Observational Research. PloS one. 2016;11(2):e0149144 10.1371/journal.pone.0149144 26886098 PMC4757561

[6] Smith TW, Hout M, Marsden PV. General Social Survey, 1972–2012 [Cumulative File]. Inter-university Consortium for Political and Social Research (ICPSR) [distributor]; 2013.

